# 

**Published:** 1961-07

**Authors:** 


					THE
MEDICAL JOURNAL
OF THE
SOUTH-WEST
Incorporating
The Bristol Medico-Chirurgical Journal
July 1961
76 (iii). No. 281 Price 4/-

				

